# Urinary mRNA-based biomarkers for non-muscle-invasive bladder cancer: a mini-review

**DOI:** 10.3389/fonc.2024.1441883

**Published:** 2024-08-09

**Authors:** Karoline Brito Caetano Andrade Coelho, Denise Kusma Wosniaki, Anelis Maria Marin, Laura Fabris, Rodolfo Borges dos Reis, Mateus Nóbrega Aoki, Dalila Lucíola Zanette

**Affiliations:** ^1^ Uro-Oncology Laboratory, Surgery and Anatomy Department, Ribeirao Preto Medical School, University of Sao Paulo, Ribeirão Preto, São Paulo, Brazil; ^2^ Laboratory for Applied Science and Technology in Health, Carlos Chagas Institute, Oswaldo Cruz Foundation (Fiocruz), Curitiba, Paraná, Brazil; ^3^ Department of Applied Science and Technology, Politecnico di Torino, Torino, Italy

**Keywords:** non-muscle invasive bladder cancer (NMIBC), urinary biomarkers, mRNA-based, muscle-invasive bladder cancer (MIBC), surveillance, aggressiveness

## Abstract

Bladder cancer (BC) is the second most common type of cancer of the urinary system. Approximately 75% of the cases are non-muscle invasive bladder cancer (NMIBC), which has a high recurrence and progression rate. Current diagnosis and surveillance methods present challenges, including risks to the patients. For this reason, urinary biomarkers have been proposed as alternatives to the methods. The goal of this mini-review is to describe urinary mRNA-based biomarkers available in current literature for NMIBC tumors, using the PubMed database. The search included the following keywords: “biomarkers” AND “bladder cancer” AND “urine” and “RNA” and “non-muscle”. The search yielded 11 original researchers utilizing mRNA-based urinary biomarkers. Although there is a wide variety of biomarkers described, the cohorts of the studies were not exclusively NMIBC, which is the subtype of BC that would mostly benefit from the introduction of a good follow-up biomarker, highlighting the need for randomized interventional trials for NMIBC.

## Introduction

Bladder cancer (BC) is the second most common type of cancer of the urinary system and the thirteenth most common cause of cancer death worldwide (https://gco.iarc.fr/). Although there are several risk factors for BC, 82% of all cases are due to modifiable risk factors (lifestyle and occupational exposure). Tobacco is the most recognized risk factor for BC (1).

Over 90% of BC cases are classified as Urothelial Cell Carcinoma (UCC) or Transitional Cell Carcinoma (TCC), which originates from the urothelium of the bladder. UCC can be subdivided into non-muscle-invasive (NMIBC), muscle-invasive (MIBC), or metastatic. Approximately 75% of all cases present the subtype NMIBC, while 25% have MIBC or metastatic disease. Tumors that remain confined to the epithelium (urothelium) are defined as NMIBC (Stages Ta, T1, and Tis), and the tumors that invade the muscle layer of the bladder, (Stage T2), perivesical fat (Stage T3), or the adjacent organs (Stage T4) are defined as MIBC (2–4).

Cystoscopy is the gold standard for the diagnosis and surveillance of BC (5, 6) Patient with non-invasive disease (NMIBC) present a higher risk of recurrence and progression to muscle-invasive disease and their follow-up requires a greater frequency of cystoscopies. Nevertheless, cystoscopy is an invasive exam for the patient and expensive for public health systems. In addition, it may generate infection, pain, and, in some cases, hematuria (4, 7). Urinary cytology can be useful as a noninvasive, inexpensive, and highly specific tool to complement cystoscopy. Cytology has a moderate sensitivity to detect high-grade lesions, but its sensitivity is low, around 20 to 50% for low-grade papillary tumors (8). As a result, most patients with a cytologic diagnosis of a low-grade urothelial neoplasm prove not to have a tumor. The false-positive rates of urine cytology range from 1.3% to 15%, and false positives occur in patients with bladder stones, human polyomavirus infections, and prior chemotherapy (9).

For this reason, non-invasive, ancillary tools that allow a longer interval between cystoscopies are needed to reduce risks to the patients and costs to the healthcare systems, especially in the case of NMIBC tumors. Based on this rationale, urinary biomarkers for detection and surveillance have been proposed for decades as alternatives to cystoscopy. The urine is a perfect candidate as a biological sample, not only because it is obtained in a non-invasive way, but also because of its continuous contact with the bladder tumoral tissue, which enables it to provide useful transcriptomic, epigenetic, and genomic insights that may be related to BC (10) In this mini-review, we will focus on describing of urinary mRNA-based biomarkers to identify potential biomarkers to identify aggressiveness in NMIBC.

## Urinary biomarkers

Studies have proposed urinary biomarkers for the detection and surveillance of bladder cancer including urine cytology, protein-based, cell-based, genomic, and transcriptomic approaches. Cell and protein-based biomarkers have been approved by the Food and Drug Administration (FDA), such as Cytology, uCyt+, and UroVysion (exfoliated cells in urine sample) and NMP22 enzyme and Bladder Tumor Antigen – BTA – (protein in urine sample) (11). Although they show increased sensitivity for low-grade tumors, their specificity still doesn’t surpass cytology. Currently, the recommendation in the European Association of Urology (EAU) guidelines continues to be cytology, in association with cystoscopy.

Despite substantial efforts, there is still a need for randomized interventional trials that are multicentric and prospective. The currently available literature is still discrepant, with small cohorts, usually performed in only one center, with analytics divergences, with the result that the biomarkers that have been identified to date do not present superior accuracy to the gold standard. Because of these limitations, these biomarkers have not been incorporated into current clinical practice (12).

Numerous reports have proposed proteins, DNA or RNA biomarkers for diagnosis of BC in urine. Until the year 2000, protein biomarkers were dominant in the literature, but more recently the proportion of DNA and RNA studies has increased (13). Protein-based biomarkers are susceptible to conditions in which the presence of protein is increased in the urine, such as inflammation, hematuria, and kidney stones. DNA-based biomarkers assess genetic alterations (point mutations, copy number alterations, and epigenetic changes including DNA methylation). The stability of the DNA molecules is an advantage over messenger RNA since the collection and transportation of samples would be simpler for DNA, but mRNA has great potential, so it has been recently described in an increasing number of studies. Compared with protein biomarkers, RNA biomarkers can be detected with greater sensitivity and specificity, and in general primers and probes are cheaper than antibodies, which are used to detect proteins. There are several possible RNA biomarkers, both coding and non-coding RNAs, including microRNAs, long non-coding RNAs, and circRNAs, which have been studied as potential biomarkers in bladder cancer in the past few years (11).

Studies using extracellular vesicles still present many challenges in clinical practice due to the lack of standardization in the methodologies for isolation and analysis and the lack of multicentric validations, despite all the efforts from the scientific community to standardize methods and results in this area (14).

mRNAs have advantages over protein and DNA biomarkers that compensate its instability that requires special conditions of collection and transportation since the methods to detect mRNA biomarkers have lower costs when compared to protein biomarkers and provide dynamic insights into cellular states and regulatory processes compared to DNA biomarkers (15). This review describes the utility and accuracy of messenger RNAs as biomarkers to monitor NMIBC by evaluating differentially expressed transcripts present in cell-free urine or urine cells (**Supplementary Figure 1**).

## Potential mRNA-based urinary biomarkers for NMIBC

### 5-mRNA (*ABL1, ANXA10, UPK1B, CRH*, and *IGF2*)

Pichler and colleagues (2018) (16) analyzed the 5-mRNA (*ABL1, ANXA10, UPK1B, CRH*, and *IGF2*) model proposed by Wallace and colleagues (2018) (17), now named Xpert BC Monitor and showed that it presents sensitivity superior to cytology, even in NMIBC low-grade and pTa disease, while overall specificity is similar. Xpert BC Monitor successfully discriminated between tumor stages, grades, size, and number of tumors, and previous intravesical instillations didn’t increase the rate of false positivity. In addition, combining this test with barbotage cytology (bladder washing) did not enhance diagnostic accuracy compared with the test alone (AUC=0.85 vs. AUC=0.87). In contrast, a prospective study with 230 patients with NMIBC tumors showed that overall sensitivity for Xpert BC Monitor was higher than for cytology and when combined, Xpert BC Monitor and cytology, it was superior to cytology alone. However, the overall specificity for cytology is better (18).

Another report showed that Xpert had an overall high diagnostic capability to detect residual tumors in repeat biopsy after initial complete Transurethral Resection of Bladder Tumor (TURBT) of T1BC (Stage T1 of BC) in NMIBC patients, with a sensitivity of 86% and negative predictive value (NPV) of 89%. The results of the Xpert test were independently associated with early tumor recurrence, suggesting that Xpert can detect genetic abnormalities before macroscopic existence by checking cystoscopy (19). The approach could help reduce invasiveness in follow-up of these patients due to the partially reduced need for cystoscopy and, consequently, could improve adherence. In concordance, other studies have described that Xpert could also be a promising tool in follow-up of recurrent NMIBC patients and could function as a predictive tool to determine the presence of residual tumors after primary TURBT. The Xpert Monitor presented higher sensitivity and an improved NPV when compared with UroVysion and cytology in patients under follow-up for BC. The specificity was minimally improved compared with UroVysion and was lower compared with cytology. Xpert was more sensitive for both high-grade and low-grade BC. The high NPV for high-grade disease is particularly important for NMIBC monitoring, as high NPV gives high confidence that the test is truly negative, allowing to reliably exclude recurrent disease. This reliability would allow waiving one cystoscopy if the Xpert result is negative, as the currently advised follow-up schedule for low-risk NMIBC consists of cystoscopy at three and twelve months after TURBT. Additionally, Xpert showed robust reproducibility and good specificity in non-BC patients (18).

Briefly, the biomarkers found in the Xpert BC Monitor test are mRNAs translated into proteins that are related to cell pathways such as cell division, adhesion, differentiation, and response to stress (*ABL1*), cell growth and signal transduction (*ANXA10*), epigenetic dysregulation in BC (*UPK1B*), neuroendocrine stress response, immunity, and inflammation (*CRH*), and proliferation and survival (*IGF2*) (16).

ABL proto-oncogene 1, non-receptor tyrosine kinase (*ABL1*) encodes a protein tyrosine kinase involved in a variety of cellular processes. The BCR region of ABL1 presents retrotransposon repeats that have been associated with bladder cancer (20). Annexin A10 (*ANXA10*) encodes a member of the annexin family of calcium-dependent phospholipid-binding proteins. This protein was found to play a role in the regulation of cellular growth and signal transduction pathways in BC. *UPK1B* encodes a uroplakin. Four different uroplakin proteins are known at present. These proteins heterodimerize and form urothelial plaques on the surface of urothelial cells. Uroplakins are significantly downregulated during urothelial transformation and tumorigenesis. In BC, *UPK1B* gene transcription is regulated epigenetically via CpG methylation. The corticotropin-releasing hormone (*CRH*) system was initially identified as a hypothalamus-directed mediator of neuroendocrine stress response, while recent studies suggest a link between *CRH* and the development of solid cancers. Preclinical studies showed the proinflammatory and procarcinogen nature of CRH family peptides and their receptors, and the fact that they modulate immunity, inflammation, and tumor cell growth. The last gene in this panel, Insulin-like growth factor 2 (*IGF2*) is a mitogenic peptide hormone overexpressed in aggressive tumors and during embryonic development. The binding of *IGF2* to its receptor, IGF1R, initiates breast and lung tumorigenesis and promotes the progression of endometrial and gastric cancers. Overexpression of *IGF2* is at least partly caused by loss of imprinting in prostate, and colon cancers, but its deregulation may also be attributable to an abnormal expression of transcription factors. Thus IGF2/IGF1R signaling enhances tumor progression in several cancers (21), but its contribution to BC progression is still unclear despite its good performance as one of the biomarkers of the XPert BC Monitor test.

According to these authors, the limitation of the use of mRNA-based techniques is the difficulty of obtaining enough high-quality RNA from voided urine. In the Xpert test, the *ABL1* mRNA functions as a sample adequacy control to verify that the sample contains human cells and human RNA. Moreover, there are some discrepancies between the studies utilizing Xpert: 1) variability in sensibility (85.9%, 84%, and 46%), 2) variability in specificity (72.3%, 91%, and 77%), and 3) lack of validation (**Table 1**). Although promising results, the test accuracy was discrepant between the studies, and research on long-term follow-up is needed.

**Table 1:** Original researchers utilizing mRNA-based biomarkers.

References	Year	Only NMIBC	NMIBC sample	Markers	Urine use (Isolation RNA)	Method	Recurrence (%)	SN^a^ (%)	SP^b^ (%)	NPV^c^ (%)	PPV^d^ (%)	AUC^e^	Validation	Aim
PMID: 33785220 (19)	2021	Yes	NMIBC, n=254	*ABL1, ANXA10, UPK1B, CRH*, and *IGF2*	Xpert Urine Transport Reagent Kit	RT-PCR	24	85.9	72.3	88.9	66.4	0.78	No	Recurrence
PMID: 28941000 (16)	2018	Yes	NMIBC, n=140	*ABL1, ANXA10, UPK1B, CRH*, and *IGF2*	Xpert Urine Transport Reagent Kit	RT-PCR	NA	84.0	91.0	93.0	NA	0.87	No	Surveillance
PMID: 30553612 (18)	2019	No	–	*ABL1, ANXA10, UPK1B, CRH*, and *IGF2*	Xpert Urine Transport Reagent Kit	RT-PCR	18	74	80	93	27.8	NA	No	Recurrence
PMID: 30355587 (22)	2019	Yes	NMIBC, n=230	*ABL1, ANXA10, UPK1B, CRH*, and *IGF2*	Xpert Urine Transport Reagent Kit	RT-PCR	22	46.2	77.0	83	36.9	0.65	No	Diagnosis
PMID: 29061538 (17)	2018	No	NMIBC, n=49	*ABL1, ANXA10, UPK1B, CRH*, and *IGF2*	Xpert Urine Transport Reagent Kit	RT-qPCR	NA	73.0	NA	NA	NA	0.87	Yes	Diagnosis
PMID: 27986532 (23)	2017	No	–	*CDC2, HOXA13, MDK, CXCR2, and IGFBP5*	Not reported		NA	92.0	97.0	0.96	NA	0.66	NA	Surveillance
PMID: 28366272 (24)	2017	No	NMIBC, n=957	*CDC2, HOXA13, MDK, CXCR2, and IGFBP5*	The voided mid-stream urine was stabilized according to the manufacturer’s instructions for each comparator test.		NA	91.0	NA	0.96	NA	NA	No	Diagnosis
PMID: 22818138 (25)	2012	No	NMIBC, n=55	*CDC2, HOXA13, MDK, CXCR2, and IGFBP5*	Voided urine was mixed with an equal volume of Cxbladder storage buffer		NA	91.0	90.0	NA	NA	0.87	No	Risk stratification
PMID: 33766467 (26)	2021	No	NMIBC, n=59	*ROBO1, WNT5A, CDC42BPB, ABL1, CRH, IGF2, ANXA10, and UPK1B*	Not reported	RT-PCR	NA	92.5	73.5	97.4	47.1	0.923	No	Risk stratification
PMID: 30771285 (27)	2019	No	NMIBC, n=127	*ANXA10, IGF2,KIFC3,KRT20,LCN2, MAGEA3,RPS21, and SLC1A6*	Cell pellet (TRIzol reagent, Invitro)	RNA-seq and nCounter	NA	94.0	NA	98.0	NA	0.823	Yes	Diagnosis
PMID: 24852426 (28)	2014	No	NMIBC, n=50	XIAP	Pellet (RNA purification kit, Norgen Biotek)	RT-PCR	44	82.91	78.38	NA	NA	0.85	NA	Diagnosis

a Sensibility.

b Specificity.

c Negative predictive value.

d Positive predictive value.

e Area under curve.

NA, Non applicable.

## Potential mRNA-based urinary biomarkers for NMIBC and MIBC

### Cxbladder Monitor Test (*CDC2, HOXA13, MDK, CXCR2*, and *IGFBP5*)

O’Sullivan and colleagues (2012) (26) developed 2 classifiers for risk stratification of urothelial cancer from their mRNA assay data (29). The classifier Cxbladder-D included the fifth marker, neutrophil marker (*CXCR2*), to reduce the risk of false-positive results in the inflamed urothelium. The second classifier, Cxbladder-S, was able to stratify tumors into low-risk - low-grade stage Ta, with a sensitivity of 91% and a specificity of 90%, respectively. In addition, the same group showed that the quantitative measurement of these five gene expression markers presented high sensitivity and negative predictive value to rule out recurrent urothelial carcinoma during surveillance (23).

Furthermore, the CxBladder Monitor showed superior performance compared with currently available, FDA-approved urine tests used as adjuncts to cystoscopy. Subgroup analyses demonstrated superior sensitivity and NPV for Cxbladder Monitor regardless of patient age and sex, or recurrent tumor size, stage, or grade by comparison with NPM22 Elisa, NPM22 Bladder Check and cytology. CxBladder Monitor had a superior sensitivity compared to NMP22 enzyme-linked immunosorbent assay, NMP22 BladderChek, and UroVysion fluorescence and urine cytology, in patients Ta, Tis, and ≥T1 undergoing monitoring for recurrence. The clinical utility of Cxbladder Monitor was demonstrated as a confirmatory negative test that may be used as an adjunctive to cystoscopy, improving the monitoring for recurrent UC, or as a direct rule-out test for patients identified as being at low risk for recurrent disease (24). Thus, CxBladder may be useful as an adjunct tool to cystoscopy to risk stratify and monitor recurrence in patients with urothelial cancer. Moreover, a study from Li and collaborators showed that the use of CxMonitor (CxM) as a home urine test allowed patients to skip their scheduled surveillance cystoscopy in the presence of a CxM-negative test. The authors report that 66 CxM-negative patients skipped cystoscopy, and none had findings on follow-up cystoscopy that required biopsies (30).

The biomarkers of the CxBladder Monitor are distinct from those in the XPert BC test. In summary, the biomarkers found in the Cxbladder Monitor Test are mRNAs that are translated into proteins related to cell pathways such as cell cycle (*CDC2*), gene expression regulation, morphogenesis, and differentiation (*HOXA13*), migration, growth, and angiogenesis (*MDK*), and cellular response, regulation of smooth muscle cell migration and proliferation (*IGFBP5*) (https://www.ncbi.nlm.nih.gov/home/genes/). *CDC2* (CDK1) CDK1 phosphorylates TFCP2L1, a pluripotency‐associated transcription factor, and the CDK1‐TFCP2L1 pathway is activated in BC cells, stimulating their proliferation, self‐renewal, and invasion. In patients with BC, high co‐expression of *TFCP2L1* and *CDK1* was associated with unfavorable clinical characteristics including tumor grade and distant metastasis (31). *HOXA13* gene is higher in low-grade tumors compared to high-grade BC tumor samples, which suggests its potential as a diagnostic marker in NMIBC. The expression level of *HOXA13* has also been reported to be higher in NMIBC urine samples than in normal controls. *HOXA13* gene expression has been tested as a diagnostic marker for NMIBC along with *PLK1* and *FGFR3* by Valizadeh and colleagues, who describe *HOXA13* with a greater sensitivity compared to *PLK1* and *FGFR3* (32). Midkine (*MDK*) is a heparin-binding growth factor that is overexpressed in bladder tumor tissue and urine from BC patients when compared to healthy individuals. It has been shown that microscopic hematuria and infection were not obstacles to detecting BC by *MDK* mRNA test (PMID: (33). IGFBP5 prolongs insulin-like growth factors (IGFs) half-life and restricts their function, affecting the IGF signaling pathway, which plays a role in cellular growth, differentiation, and apoptosis. IGFBP5 overexpression strongly correlates with several adverse prognostic factors in BC (34).

### 3-marker urinary panel (*ROBO1, CRH*, and *IGF2*)

A 3-marker urinary mRNA panel was proposed by Shkolyar and colleagues (2021) (26) to identify intermediate and high-risk BC patients undergoing surveillance. The *ROBO1*, *CRH*, and *IGF2* gene expression levels were associated with increased risk with a sensitivity of 92.5% and specificity of 73.5%. This panel consists of two genes that were already included in Xpert BC (*CRH* and *IGF2*), while *ROBO1* was included for the first time in a panel of biomarkers of BC. Robo1 protein (ROBO1) is overexpressed in human bladder cancer tissues and paracarcinoma tissues (35). Despite this being a promising panel, the study included a small cohort in a single center, therefore further validation is needed.

### x8-gene expression classifier (*ANXA10, LCN2, KRT20, SLC1A6, RPS21, IGF2, MAGEA3*, and *KIFC3*)

This panel was developed in serial steps, from the discovery to the validation phases, performed by the same group and in a multicentric international cohort. Logistic regression analysis was used to generate an 8-gene expression classifier (*ANXA10, IGF2, KIFC3, KRT20, LCN2, MAGEA3, RPS21*, and *SLC1A6*) that showed an area under the curve (AUC) of 0.893 for detecting BC. The 8-gene classifier was also tested in an independent multicentric, international cohort composed of patients in follow-up for BC. The 8-gene classifier performed equally in all BC risk groups, with high and comparable overall sensibility in low-grade and high-grade tumors. The authors reported that their 8-gene expression classifier outperforms the current gold standard (cystoscopy) as well as the previously developed gene expression tests in terms of sensitivity and negative predictive value. It was reported that by using the classifier, around 17% of BC patients under follow-up in their validation cohort could safely skip cystoscopy, while the remaining patients should undergo cystoscopy. The 8-gene classifier is described as safe to guarantee the detection of potential life-threatening tumors in cases of high-risk NMIBC, due to its high sensitivity and NPV (27). *MAGEA3* is a cancer-testis antigen that has been reported to be overexpressed in 15% of the patients with BC by immunohistochemistry. Kaplan-Meier analysis revealed significantly worse 5-year progression-free survival associated with a strong expression of MAGEA3 (36) *LCN2/MMP-9* pathway has been associated with an aggressive phenotype of bladder cancer and the elevated NPV of this protein complex makes them candidate markers of exclusion test for bladder cancer (37). Up-regulated expression of *KIFC3* has been described in many types of cancer and is associated with Epithelial-Mesenchymal-Transition and other important events in tumor development and progression (38) *KRT20* (cytokeratin 20) gene was selected as a surrogate marker (along with 3 uroplakin genes) for luminal MIBC subtype by Olkhov-Mitsel and colleagues in the analysis performed with tumoral tissue (39). To our knowledge, *RPS21* was not previously reported in BC, except from the 8-gene classifier described by Montalbo and colleagues.

### X-linked inhibitor of apoptosis (*XIAP*)

The X-linked inhibitor of apoptosis (*XIAP*) is an IAP protein family member that acts as an inhibitor of the caspase/apoptosis pathway. Urinary *XIAP* gene expression was investigated as a biomarker in BC by Srivastava and colleagues (2014) (28). These authors demonstrated a better sensitivity for *XIAP* gene expression in primary NMIBC cases when compared with voided urine cytology. However, the same study showed that *XIAP* had lower sensitivity than the cytology for recurrent cases. *XIAP* was more sensitive than cytology for the diagnosis of BC patients with early stage. Similarly, a better sensitivity of *XIAP* was detected for both higher- and lower-grade TCC cases as compared to cytology. Expression significantly correlated with tumor grade in this study, but the authors discuss the lack of previous evidence (28).

## Discussion

**Figure 1:**
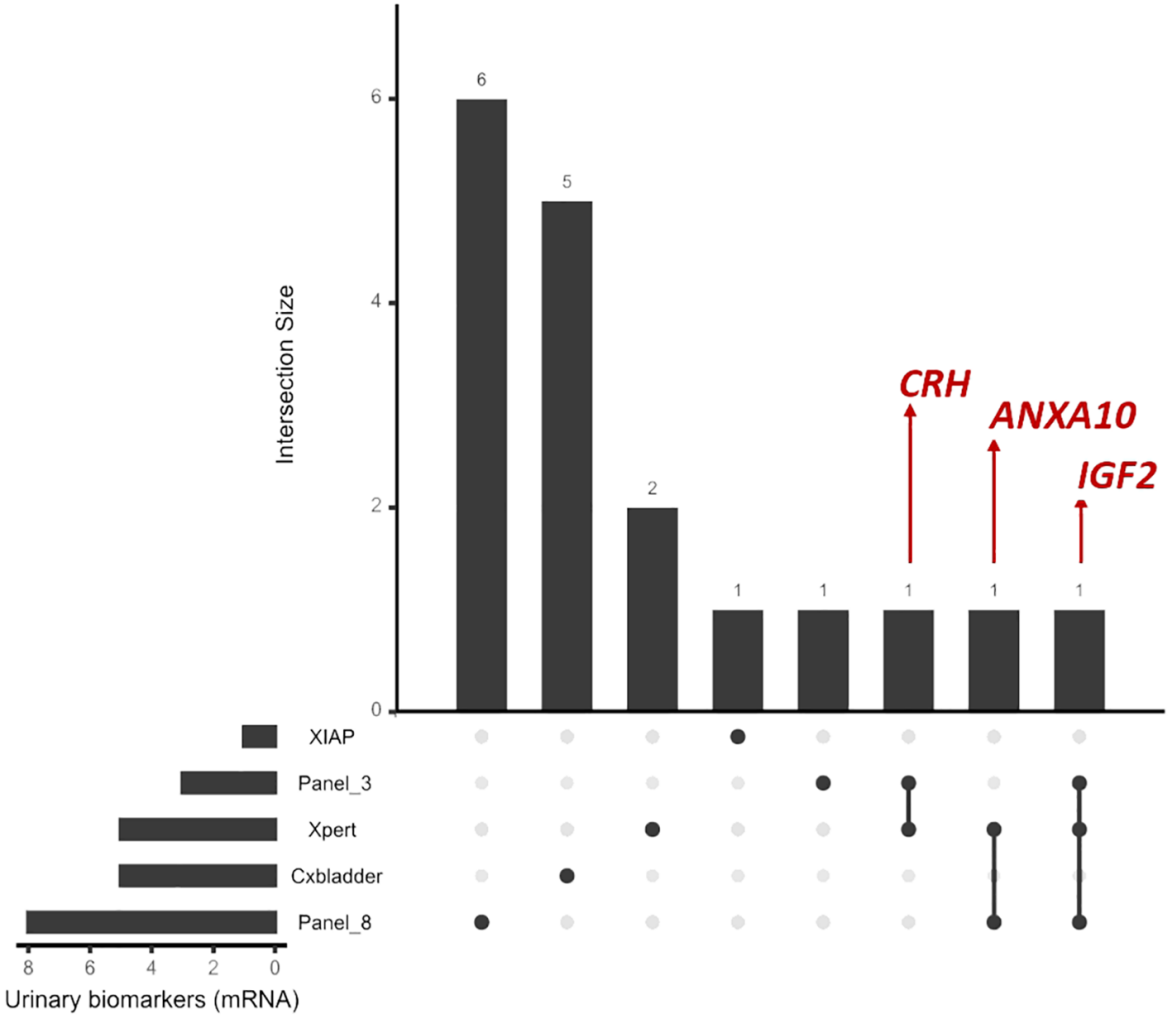
Visualization of the intersection between the markers (mRNA) of the tests described using the UpSetR package in R.

The mRNA urinary panels of BC markers described here have exclusive and shared markers. Among the shared markers, we highlight the presence of *IGF2* in the Xpert, in the 3-marker urinary panel (Panel_3), and the x8-gene expression classifier (Panel_8), The *CRH* gene is part of the Xpert and 3-marker urinary panel. Similarly, the *ANXA10* gene is part of two panels, the Xpert and x8-gene expression classifier. The other genes are mutually exclusive between panels (**Figure 1**). Although the 3-marker urinary panel and x8-gene expression classifier had mixed cohorts (MIBC and NMIBC tumors), they contain markers that were present in the previously mentioned Xpert test, although two of the genes found in the Xpert Test are exclusive (*ABL1* and *UPK1B*). While *CRH* is shared with the 3-marker urinary panel, *ANXA10* is shared with the 8-gene expression classifier, and *IGF2* is shared with the two panels (**Figure 1**).

The high rate of recurrence in NMIBC requires follow-up with cystoscopies every three months for two years, and subsequently in more spaced intervals. Moreover, cystoscopy is an invasive procedure, associated with some risks, while urine cytology is inexpensive but has a limited performance for low-grade tumors. These are the main reasons that justify the search for more efficient and less invasive biomarkers for the surveillance of NMIBC. The ideal biological sample would be urine, given its non-invasive nature. Despite several efforts and studies seeking an ideal panel of urinary biomarkers, there is no consensus.

As previously discussed, only three studies included cohorts exclusively of NMIBC, which makes a more comprehensive review difficult. However, we described biomarkers available in the current literature for both types of BC, reinforcing the need for validation and new studies for patients with NMIBC tumors. Urine is the biological sample that is the most reliable and non-invasive source of biomarkers in bladder cancer, as well as in other urological malignancies since the tumor mass is in close and direct contact with urine. This makes urine a liquid biopsy sample but also a source of exfoliated cancer cells. NMIBC has a high risk of recurrence and progression to muscle-invasive disease, requiring follow-up with repeated cystoscopies, which are invasive and expensive. This is the main reason for the extensive research to find new biomarkers and improve those that are already described. Good biomarkers to evaluate the diagnosis and progression of bladder cancer would facilitate follow-up and increase the quality of life of BC patients. There are a lot of unexplored possibilities to be studied for the discovery and validation in this field, so this mini-review describes the existing panels of mRNAs that act as biomarkers in bladder cancer, with a special focus on NMIBC.
