# Supplementary material for: Urinary mRNA-based biomarkers for non-muscle-invasive bladder cancer: a mini-review
Source: Front Oncol. 2024 Aug 9;14:1441883. doi: 10.3389/fonc.2024.1441883 (PMC11341357; doi:10.3389/fonc.2024.1441883)
Supplement: Supplementary file 1 [file DataSheet_1.pdf]

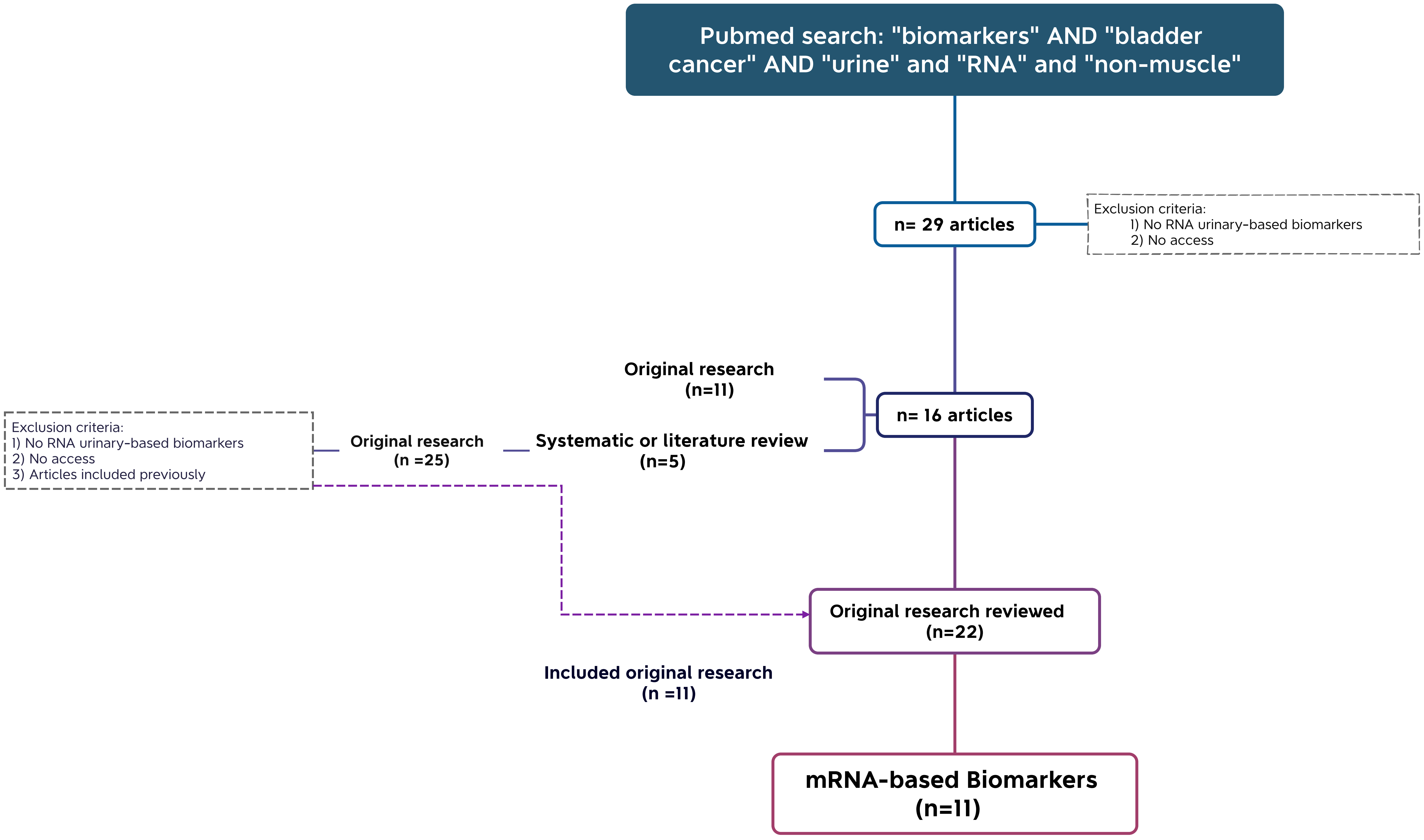

Presented with xmind
